# Neuroendocrine tumors of the urinary bladder – Report of two cases

**DOI:** 10.4322/acr.2021.305

**Published:** 2021-08-20

**Authors:** Meenakshi Rao, Apoorvi Dubey, Himanshu Pandey, Binit Sureka, Pawan Kumar Garg, Harishkumar Bohra, Poonam Elhence

**Affiliations:** 1 All India Institute of Medical Sciences, Department of Pathology and Lab Medicine, Jodhpur, Rajasthan, India; 2 All India Institute of Medical Sciences, Department of Urology, Jodhpur, Rajasthan, India; 3 All India Institute of Medical Sciences, Department of Radiodiagnosis and interventional Radiology, Jodhpur, Rajasthan, India

**Keywords:** Paraganglioma, Carcinoma, Small Cell, Urinary bladder neoplasms

## Abstract

Primary paraganglioma and small cell neuroendocrine carcinoma of the urinary bladder are rare tumors, comprising 0.05% of all bladder tumors and <1% of all malignant bladder tumors, respectively. These tumors can be the cause of a diagnostic dilemma or misdiagnosis on morphology. Paraganglioma is often mistaken for urothelial carcinoma and small cell carcinoma for poorly differentiated carcinoma or lymphoma. Herein, we report a case of primary paraganglioma and another of a small cell carcinoma of the urinary bladder and discuss their closest differential diagnoses. The diagnostic pitfalls should be kept in mind so that correct, timely diagnosis of these entities can be made due to implications in the management and prognosis.

## INTRODUCTION

Primary neuroendocrine neoplasms of the urinary bladder are very rare. The current WHO/ISUP classification (2016) recognizes four distinct neuroendocrine neoplasms of urinary bladder – (i) Small cell neuroendocrine carcinoma (SmCC), (ii) large cell neuroendocrine carcinoma, (iii) well-differentiated neuroendocrine tumor, and (iv) paraganglioma.^1^

Paragangliomas comprise 0.05% of all bladder tumors, and approximately 10% occur in extra-adrenal paragangliomas.^1^ They arise from chromaffin tissue of the sympathetic nervous system and can be functional or non-functional. Patients with functional paragangliomas can have headache, palpitations, fever, diaphoresis, and rare symptoms like visual blurring, flushing, vomiting, dyspnea, and dizziness.^2^ Patients with non-functional paragangliomas often present with non-specific complaints, are clinically unsuspected and may lead to erroneous diagnoses.

SmCC accounts for <1% of malignant bladder tumors. The pathogenesis of these tumors is controversial. The various proposed theories suggest these tumors arise from urothelial carcinoma in which the tumor cells dedifferentiate, or from the neuroendocrine cells in the epithelium and less likely from the urothelial stem cells. These high-grade malignancies also cause non-specific clinical features like gross hematuria, dysuria, obstruction and are also clinically unsuspected. Metastasis is common at presentation.

Herein, we report two clinically unsuspected and misdiagnosed primary neuroendocrine neoplasms of urinary bladder- Paraganglioma and SmCC, with a brief discussion of their diagnostic pitfalls.

## CASE 1

**Figure 1:**
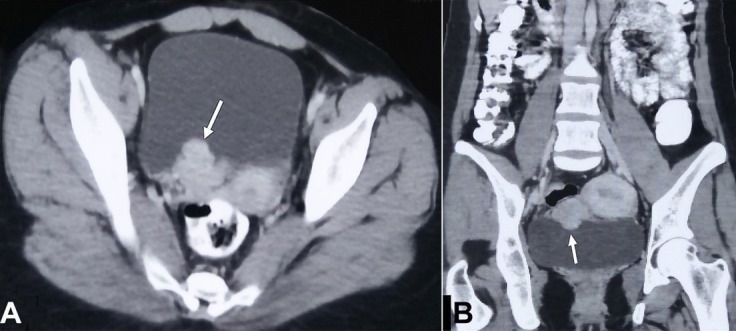
**A** – Axial; and **B** – Coronal contrast enhanced CT abdomen reveal nodular, polypoidal enhancing mass (white arrow) arising from right posterior wall of urinary bladder.

A 21-year-old woman was referred to the Urology Department for management of a high-grade invasive urothelial carcinoma of the urinary bladder, reported on a biopsy performed outside. The only morphological examination was done, and immunohistochemistry (IHC) had not been performed. She had a single episode of gross painless hematuria one month back, for which she had been evaluated. On evaluation, she was asymptomatic, with no remarkable physical examination. Her pulse was 76/min, blood pressure was 110/70mm Hg, and respiratory rate was 18/min. Her blood and urine workup were within normal limits. The abdominal and pelvic contrast-enhanced computed tomography (CT) revealed a nodular and polypoidal enhancing mass from the right posterior wall of the urinary bladder (Figure 1).

**Figure 2:**
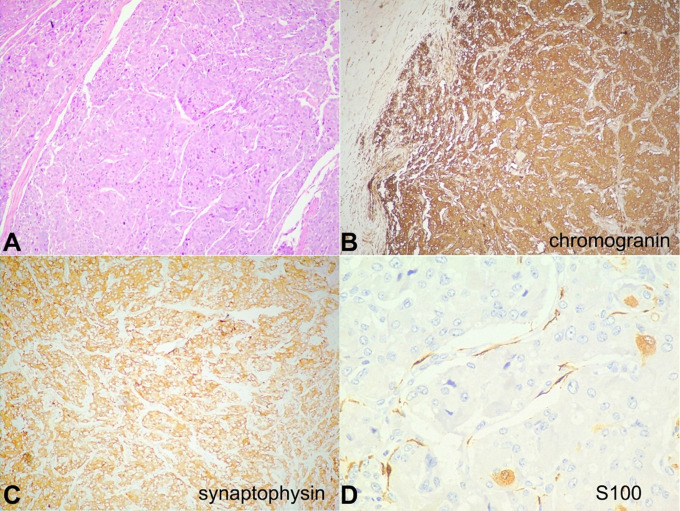
**A** – Photomicrograph showing a tumor arranged in nests and trabeculae separated by thin fibrovascular septae, with ‘Zellballen pattern’ (H&E 100X); **B** – On IHC, the tumor cells are positive for chromogranin (100X); and **C** – synaptophysin (100X); **D** – S100 protein is expressed by sustentacular cells at the periphery of the cell nests (400X).

The initial biopsy was reported elsewhere as a high-grade invasive urothelial carcinoma, and the blocks and slides were reviewed. Sections showed the bladder, focally lined by urothelial lining, with a tumor in the lamina propria, arranged in nests and trabeculae separated by thin fibrovascular septae, with ‘Zellballen pattern’ (Figure 2A). The tumor cells were large, with moderate to abundant amounts of eosinophilic granular cytoplasm, round to oval nuclei, stippled chromatin, and inconspicuous nucleoli. Mitoses were <2/10 high power fields, and necrosis was not identified. The deep muscle was not included in the biopsy. On immunohistochemistry (IHC), the tumor cells were diffusely and strongly positive for chromogranin and synaptophysin. S100 protein was positive in the periphery of the nests in the sustentacular cells (Figures 2B-D). The tumor cells were negative for CK7, CK20, and p63, ruling out a urothelial neoplasm. Ki-67 proliferation index was <2%. After review, a final diagnosis of paraganglioma was made. Urinary and plasma nor-metanephrine, metanephrine levels, and urinary VMA levels were within normal limits. Patient was advised for an MIBG scan/ Gallium 68 DOTANOC PET/CT scan; however, it was not done due to affordability issues.

No other lesion was found on a routine radiology scan (CT scan). Following the biopsy report, a partial cystectomy was received, which showed similar features. The patient is on follow-up (12 months to date) and doing well.

## CASE 2

**Figure 3:**
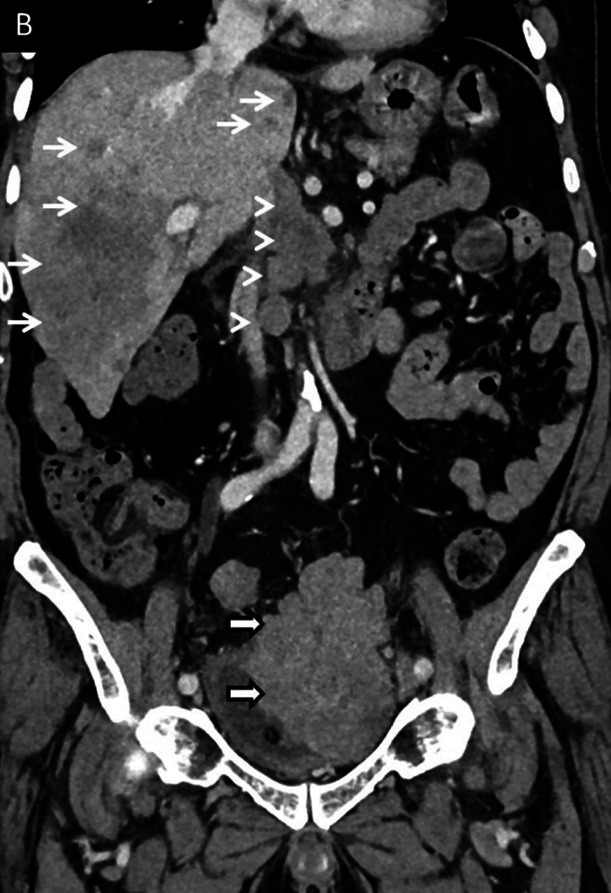
**B** – Coronal contrast-enhanced CT images arterial phase showing lobulated enhancing bladder mass (thick arrows), venous phase CT images showing paravesical extension (black arrows) with bulky retroperitoneal and abdominal lymphadenopathy (arrowheads) and multiple liver metastases (white arrows).

An 81-year-old man was referred to the Urology Department to manage a high-grade, poorly differentiated carcinoma of the urinary bladder, reported on a biopsy performed outside. He had a history of repeated episodes of gross hematuria, weight loss, and reduced appetite. On evaluation, he was found to be cachexic, with pallor and icterus. He was anemic, with raised bilirubin and mildly altered liver enzymes. The contrast-enhanced CT scan showed a lobulated enhancing bladder mass with a paravesical extension (Figure 3), abdominal lymphadenopathy and multiple liver metastases

**Fig. 4:**
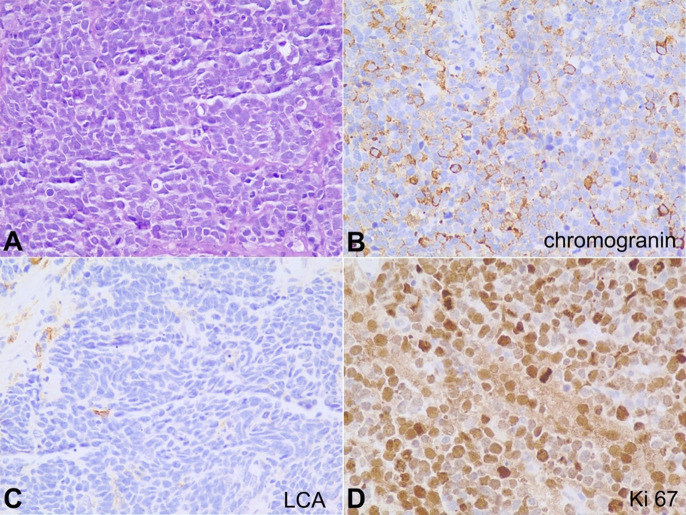
**A** – Photomicrograph showing a tumor with cells having high nuclear-cytoplasmic ratio and hyperchromatic nuclei arranged in diffuse sheets, H&E 400X; **B** – The tumor cells show partial cytoplasmic granular positivity for chromogranin, 400X; **C** – The tumor cells are negative for LCA, 400X; **D** – The Ki-67 proliferation index of the tumor is very high, 99% (400X).

. The initial biopsy was reported elsewhere as poorly differentiated carcinoma. IHC was not performed on the initial biopsy. The slides were reviewed. Sections from the lesion showed a proliferation of tumor cells arranged in sheets and nests, with moderately pleomorphic small cells, with scant cytoplasm, hyperchromatic round to overlapping oval nuclei, and inconspicuous nucleoli (Figure 4A). Brisk mitoses (>50 mitotic figures/10HPF) and a few karyorrhectic debris were noted. Hemorrhage and necrosis were also seen. On IHC, the tumor cells were positive for neuroendocrine markers, chromogranin (Figure 4B), and synaptophysin. The tumor cells also expressed positivity for EMA and p63. The tumor cells were negative for LCA, ruling out the differential diagnosis of lymphoma (Figure 4C). Based on these findings, a final diagnosis of SmCC was given. Ki-67 labeling index was 99% (Figure 4D). The patient was advised on the prognosis and started on palliative care. He is currently on follow-up (seven months to date).

## DISCUSSION

Neuroendocrine neoplasms of the urinary bladder are sporadic and often cause a diagnostic dilemma. Paraganglioma of the urinary bladder is a very rare tumor with characteristic histologic and immunohistochemical features. However, it may be misdiagnosed as urothelial cancer because: (i) the urothelial carcinomas are much more common, (ii) of the involvement of the muscularis propria, (iii) of the morphological resemblance to urothelial carcinoma in transurethral resection specimens, especially due to artifactual changes by surgical cautery, and (iv) of the failure of pathologists to consider it in the differential diagnosis while evaluating a bladder tumor due to its rarity.^2^ Furthermore, functional tumors that might suggest consideration of the diagnosis, are not always present. Seventeen percent of the paragangliomas are non-functional.^3^ Distinguishing paraganglioma from urothelial carcinoma is extremely important because of the differences in management and prognosis.^4^ The two entities can be differentiated on IHC. The paragangliomas are positive for neuroendocrine markers like chromogranin and synaptophysin and negative for epithelial markers CK7 and CK20, while the reverse profile is seen in urothelial carcinomas.

Primary small cell neuroendocrine carcinomas are also very rare bladder tumors, comprising only 0.5%-1.0% of primary bladder malignancies. The pathogenesis and origin are uncertain; they are thought to arise from the divergent differentiation in urothelial carcinoma or from urothelial stem cells.^5^ In contrast to small cell carcinoma of the lung, SmCC is only rarely associated with paraneoplastic syndromes.^6^ Accurate diagnosis is important because of implications in management and prognosis. The response rate to chemotherapy is high, but the overall prognosis is poor.^7^ Metastasis is common at presentation. The common sites of metastasis are the regional lymph nodes, bone, liver, and lung.

SmCC may be misdiagnosed as poorly differentiated urothelial carcinoma and non-Hodgkin lymphoma (NHL). Since all these three entities have different management and prognosis, differentiation between them is very important. Poorly differentiated urothelial carcinoma may also present as sheets of high-grade tumor, with necrosis and brisk mitoses. IHC for neuroendocrine markers (chromogranin, synaptophysin, and CD56) can distinguish between the two entities. SmCC can be distinguished from NHL by a panel of antibodies. Positivity for neuroendocrine markers and negativity for LCA favors a diagnosis of SmCC. Metastasis from the lung, though extremely uncommon, also has to be excluded. It is important to keep in mind that thyroid transcription factor (TTF1), considered specific for thyroid and lung carcinoma, can also be expressed in a subset of SmCC.^8^ IHC for TTF-1 was not performed in the current case, since up to 30% of small cell neuroendocrine carcinomas of the urinary bladder can show positivity for TTF-1 and there was no lesion in the lung. The salient differences between urothelial carcinoma, paraganglioma, and small cell neuroendocrine carcinoma are summarized in Table 1.

**Table 1:** Relevant features and differences between urothelial carcinomas, small cell neuroendocrine carcinomas and paragangliomas

	Urothelial carcinoma	Small cell neuroendocrine carcinoma	Paraganglioma
Age group	>50 years	>50 years	Any age, mean 43.3 years
Incidence	Most common bladder cancer, 80-90% of bladder cancers	Rare, <1% of bladder cancers	Rare, 0.05% of bladder tumors
Clinical features	Hematuria, urgency, nocturia, dysuria, Associated with smoking	Hematuria, dysuria, associated with smoking	Symptoms related to catecholamine secretion: hypertension, headache, blurred vision, intermittent gross hematuria
Morphological features	Nests, sheets, cords, trabeculae, single cells, variable cellular morphology. Well-differentiated tumors show nuclei lined up perpendicular to the basement membrane in nests. Poorly differentiated tumors may be difficult to identify as urothelial carcinomas on morphology	Sheets of small cells with hyperchromatic, overlapping nuclei and inconspicuous nucleoli	Cells arranged in distinctive nests (Zellballen), separated by delicate septa. Large, polygonal cells with smudged or hyperchromatic nuclei
IHC	Positive for epithelial markers, positive for CK7 and CK20, GATA3, p63	Positive for neuroendocrine markers, high Ki-67	Positive for neuroendocrine markers, sustentacular cells positive for S-100P
Mutation spectrum	TP53, FGFR3, PIK3CA, RB1, HRAS	RB1, genetically unstable, high numbers of genomic alterations, deletions of 10q, 4q, 5q, 13q	SDHA, SDHB, losses at 1p, 3q, 22q

In conclusion, paraganglioma and small cell neuroendocrine carcinoma are rare neuroendocrine neoplasms of the urinary bladder with unique management and prognostic implications. The diagnostic pitfalls should be kept in mind; the pathologist should be alert to the possibility of these rare diagnoses, with judicious use of IHC in suspicious cases, so that correct, timely diagnosis of these entities can be made.
